# Sexual Knowledge, Attitudes and Practices of Female Undergraduate Students in Wuhan, China: The Only-Child versus Students with Siblings

**DOI:** 10.1371/journal.pone.0073797

**Published:** 2013-09-04

**Authors:** Shiyue Li, Rucheng Chen, Yue Cao, Jingjing Li, Dan Zuo, Hong Yan

**Affiliations:** 1 School of Public Health of Wuhan University, Wuhan, Hubei, P.R. of China; 2 Globe Health Center of Wuhan University, Wuhan, Hubei, P.R. of China; University of Westminster, United Kingdom

## Abstract

**Objectives:**

This study explored sexual knowledge, attitudes and practices of female only-child undergraduates and made a comparison with students with siblings.

**Methods:**

Anonymously completed questionnaires were received from 4,769 female undergraduates, recruited using randomized cluster sampling by type of university and students' major and grade. Multivariate logistic regression was used to assess the effects of only-child on sexual knowledge, attitudes and practices among female undergraduates.

**Results:**

Of 4,769 female undergraduate students, 41.0% were only-child and 59.0% were students with siblings. Compared with students with siblings, only-child students scored higher on sex-related knowledge, were more inclined to agree with premarital sex, multiple sex partners, one-night stands, extramarital lovers and homosexuality, and were more likely to have a boyfriend and experience sexual intercourse (73.6% vs. 61.4%; 24.0% vs. 14.0%). Only-children were less likely to experience coercion at first sex and have first sexual intercourse with men not their “boyfriends” than children with siblings (3.3% vs. 6.4%; 20.7% vs. 28.8%). There were no significant differences on other risky sexual behaviors (e.g. multiple sex partners and inconsistent condom use) between the only-child students and students with siblings.

**Conclusions:**

Sexual knowledge, attitudes and some practices of only-child female undergraduates were different from students with siblings. Intervention should be designed according to different requirements of only-children and non-only-children.

## Introduction

To ease the enormous pressure of population explosion, the Chinese government started promoting and implementing “one couple one child” family planning policy nationwide in 1979 [1]. The implementation of the one-child policy lasted more than thirty years; the controlling effect of the population growth is significant [2]. It also significantly affects the size and composition of the family today. Data showed that the form of the traditional extended family (three generations living under one roof) in 1982 accounted for 24.3% of urban households in China, and by 1994, this proportion dropped to 18.0%; accordingly, family model of three members showed an increasing trend in the Chinese family structure [3], especially in one-child families, the proportion of a family of three was up to 80% [4].

Family composition and size determine the mode of interaction between family members; compared with non-only-child families, the parents of only-child tend to put more time and effort on the only child and therefore undoubtedly increase the direct contact opportunities between parents and children, plus that lack of brothers and sisters during children growing process; all of which enable parents play the role of caregivers, educators, while also acting as siblings or peers. The diversity of the roles of the parents make close parent-child relationship easily form, which directly affect the child's psychological characteristics of personality and social behavior orientation; therefore, it is generally believed that the family environment inevitably make the only-child groups different from those children with siblings [4], [5].

In 2007, China Population Development Strategy Research Group pointed out in their report that China today has a total of nearly 100 million of only-child [6]; can such a large child population grow up normally, healthily and smoothly? This is a topic related to a series of major issues in China's social progress and development, and caused widespread concern from academia and society as a whole. Starting from the late 1990s, the only-child began entering university, and now this population has become the major part in various types' university [7]. In terms of the only-child college students, present researches have focused on their adaptability, personality characteristics, psychological status, values, and career and investigated students' differences by comparing those with and without siblings [8], and yet there are few literatures on topic of sexual and reproductive health status.

As a product of the times, the only-child almost synchronized with China's reform, opening up and the modernization of social development, the reform and opening up has allowed a large number of Western cultures entering China, and the rapid development of the Internet and other technology provide a convenient channel for more information [9]. In this context, China's younger generation has a much opening behavior in sexual performance than their fathers. Data showed that in 1990, Chinese college students engaged in sexual behavior's peak time was in their 20–22-year-old, while in 2000, the trend has shown a peak time of 17–19-year-old [10]. Pan Suiming's survey also found that in 1997 overall sexual intercourse rate among Chinese university students was 10.1%, and in 2006, this data has surged to 32.0% [11], and the consequences were unintended pregnancy, abortion, sexually transmitted diseases which have become a serious threat to the health of students – especially for female students – a problem that can not be ignored [12].

Affected by the one-child policy, gender preference eliminated largely in China's one-child families, the girls have the same social conditions with boys, so the girl growing up with broader development space in China's one-child families than girls in families with many children [13], resulting in the differences between the two groups in the cognitive and sexual behavior. Studies have shown that the only-child college students treat premarital cohabitation on a more open attitude than non-only-child [14], [15]; Wang Hongjing's sex status survey among 1205 female students in Chengdu also found that the differences in the proportion (17.9% vs.11.5%) of the only-child and the non-only-child in sexual activity occurred [16].

At present, China's only-child and non-only-child female students' sexual investigation were few, most of the research only involved in sexual knowledge, sexual attitudes, and sexual behavior in one or two dimensions [14]–[16]. The current study was more comprehensive, in comparison of only-child and non-only-child female students in sexual knowledge, sexual attitudes, and sexual behaviors; and tale the only-child as an independent factor to analyze its effect on female students' sexual knowledge, attitudes and behaviors, to provide guidance for carrying out targeted sex education.

## Materials and Methods

### Ethics statement

The study was approved by the Medical Ethics Committee of Wuhan University. The written consent was obtained from all students who participated in the study.

### Sample and procedures

Female college students were recruited by randomized cluster sampling in Wuhan. Of 43 universities in Wuhan, 16 were randomly selected according to type (state key universities, universities under individual Ministries, local universities). In these study sites, classes were sampled by majors and grade. The inclusion criteria of participants were (1) female college students, (2) unmarried, (3) enrolled in one of the selected classes and (4) willing to participate in the study.

The recruitment period was from December 2005 to April 2006. Investigators enrolled 5,076 female undergraduates who were unmarried and willing to participate in the study across 16 sites. A self-administered anonymous questionnaire was distributed to the students for completion in classrooms. Students were asked to put the finished questionnaire into a locked box.

Marked questionnaires were returned from 4,923 (97.0%) enrollees. All completed questionnaires were reviewed by research staff for completeness and consistency. Among initial participants, 154 questionnaires were discarded because there were large amounts of missing data. The remaining 4,769 questionnaires represented 94.0% of the initial sample.

### Measures

The questionnaire was developed for this study by reviewing relevant literature and consulting experts. Data were collected in three areas: 1) socio-demographics; 2) sex-related knowledge and attitude; 3) sex-related behavior and risks.

Socio-demographic items included age, ethnicity (Han vs. minority), major (literature and history, science and technology, medicine, or art), grade (freshman, sophomore, junior, or senior), home location (eastern coastal regions, central areas, or western areas), living place during middle school period (province capital city, county capital city or countryside), and their parents' economic states (poor, average, or rich). The students were also asked if they are only-child.

Sex-related knowledge was measured using a 27 items regarding reproduction (e.g., Do you know the stages of normal menstrual cycle?), contraceptives (e.g., Do you know how to use condom?), STDs (e.g., Is gonorrhea a sexually transmitted disease?), and AIDs (e.g., Is sexual intercourse a route of transmission of AIDs?). Response options were yes, no, and don't know; correct answers were coded 1, and other responses coded 0. Coded responses were summed and converted to a 100-point scale by multiplying by 0.037(1/27). Higher scores reflected better sex-related knowledge.

We asked students to indicate the attitudes toward premarital sex, multiple sex partners, one night stands, extramarital lovers and homosexuality. Response options were 1) approve of this behavior, 2) understand this behavior in others, and 3) disapprove of this behavior. Respondents were also asked whether condoms should be used during premarital sex (1 =  yes, 0 =  no or unclear) and whether the condom is the safest way to prevent STIs and unintended pregnancy (1 =  yes, 0 =  no).

For behavioral items, respondents were asked if they was employed in a place for entertainment, such as a pub, club and disco (1 =  yes, 0 =  no) and if they had boyfriends. Also asked were questions about masturbation. Regarding sexual behavior, respondents were asked if they ever had sexual intercourse (1 =  yes, 0 =  no). If a respondent answered “yes,” she was asked to provide more information on age at first sex, and who she had sex with during the first encounter and in the past 12 months (boyfriend, acquaintance, “one night stand” partners, or customers of sex trade, post-coded into boyfriend vs. other). She also was asked to report if she had multiple sex partners in one period (1 =  yes, 0 =  no), and her lifetime number of sexual partners. We also asked if she ever had had a sexual partner who was married, if she had been coerced during her first sex and if she had had sex with another woman.

Respondents who had ever had sex were asked if they had used a condom the first time they had sex (1 =  yes, 0 =  no). Information was also gathered about condom use in the past 12 months. Response options for the questions were ‘each time’, ‘often’, ‘seldom’ and ‘never’; responses were collapsed into two options: 1 = .never or seldom, and 0 =  often or each time. The first option was thought as inconsistent condom use.

Risks included pregnancy and reproductive infections/STDs. A respondent who had had sex was asked to report if she was ever pregnant (1 =  yes, 0 =  no) and if she had acquired any reproductive infections/STDs (1 =  yes, 0 =  no) and, if so, to name the infections (e.g., gonorrhoea, syphilis, condyloma acuminatum, etc.).

The test-retest reliability of the questionnaire has been reported in a previous article [17].

### Statistical analysis

Data were analyzed using SPSS version 13.0. Sex-related knowledge, attitudes and behaviors were analyzed by Chi-squared tests among only-children and children with siblings. Multivariate logistic regression models were used to assess the relative influences of only-children on sex-related knowledge, attitudes and behaviors.

## Results

### Sample characteristics

Table 1 shows the socio-demographic characteristics of respondents. Of 4,769 female undergraduate students, 41.0% were only-child and 59.0% were children with siblings. The mean age of students was 20.4±1.5. Respondents were distributed in each grade, the proportions of freshman, sophomore, junior and senior was 28.8%, 25.1%, 27.4% and 18.7%, respectively. A total of 39.0% of sample was majoring in literature and history, 24.5% were majoring in science and technology, 20.7% were enrolled in medical science and 15.8% were majoring in art, the number of only-child whose major was the art was larger than children with siblings (27.5% VS 7.7%). 74.4% students' home location were central areas, 13.8% were from western areas and 11.8% from eastern coastal regions. The address of respondents' middle school was divided among countryside (17.6%), county (44.1%) and province capital city (38.4%), 68.1% of only-child received secondary education in province capital cities, while the figure was only 17.8% among children with siblings. Most of students came from middle-income families (68.1%), only-child' family economic states were superior to children with siblings', 24.9% of only-child were from high-income families, while the figure were only 8.8% among children with siblings.

**Table 1:** Socio-demographic characteristics of females from one-child families and those students with siblings.

Characteristics	Total (n = 4769)	Only-child			
		Yes(n = 1954)	No(n = 2815)	*χ^2^*	*P*
**Current age**				20.034	<0.001
≤18	458(9.6)	210(10.7)	248(8.8)		
19–21	3143(65.9)	1328(68.0)	1815(64.5)		
≥22	1168(24.5)	416(21.3)	752(26.7)		
Mean ± SD	20.4±1.5	20.3±1.4	20.4±1.5		
**Major**				400.542	<0.001
Literature and history	1862(39.0)	776(39.7)	1086(38.6)		
Science and technology	1167(24.5)	323(16.5)	844(30.0)		
Medical science	985(20.7)	317(16.2)	668(23.7)		
Art	755(15.8)	538(27.5)	217(7.7)		
**Grade**				52.507	<0.001
Freshmen	1373(28.8)	492(25.2)	881(31.3)		
Sophomores	1195(25.1)	446(22.8)	749(26.6)		
Juniors	1309(27.4)	633(32.4)	676(24.0)		
Seniors	892(18.7)	383(19.6)	509(18.1)		
**Nationality**				21.290	<0.001
Han	4217(88.4)	1778(91.0)	2439(86.6)		
minority	552(11.6)	176(9.0)	376(13.4)		
**Home location**					
Eastern coastal regions	561(11.8)	221(11.3)	340(12.1)		
Central areas	3548(74.4)	1518(77.7)	2030(72.1)		
Western areas	660(13.8)	215(11.0)	445(15.8)		
**Address in your middle school**				1363.760	<0.001
Province capital city	1831(38.4)	1331(68.1)	500(17.8)		
County capital city	2101(44.1)	583(29.8)	1518(53.9)		
Countryside	837(17.5)	40(2.0)	797(28.3)		
**Parents**' **economic status**				317.018	<0.001
Poor	855(17.9)	155(7.9)	700(24.9)		
Average	3249(68.1)	1382(70.7)	1867(66.3)		
Rich	665(13.9)	417(21.3)	248(8.8)		

### Sex-related acknowledge, attitude and behavior

Table 2 showed scores in reproduction and contraception knowledge, sexually transmitted diseases and AIDS knowledge, and sexual and reproductive health knowledge were 16.0±7.3, 17.6±4.1 and 33.6±10.3 respectively, female only-child scored higher than female students with siblings. Only-child were more inclined to agree with premarital sex, multiple sex partners, one night stand, extramarital lover and homosexuality than children with siblings. 73.6% of only-child reported having a boyfriend, higher than children with siblings (61.4%), and 24.0% of only-child had sexual intercourse, while the figure was 14.0% among students with siblings.

**Table 2:** Sex-related acknowledge, attitude and behavior among students who were only-child and students with siblings.

Variables^a^	Total (n = 4769)	Only-child			
		Yes (n = 1954)	No (n = 2815)	*t* or *χ^2b^*	p
**Knowledge**					
Score in Reproduction and Contraception knowledge(Mean ± SD)	16.0±7.3	17.6±7.0	14.9±7.2	12.745	<0.001
Score in Sexually Transmitted Diseases and AIDS Knowledge(Mean ± SD)	17.6±4.1	18.3±3.9	17.2±4.2	9.474	<0.001
Score in Sexual and Reproductive Health Knowledge(Mean ± SD)	33.6±10.3	35.9±9.8	32.1±10.3	12.820	<0.001
**Attitudes**					
Agreed premarital sex	2837(59.5)	1313(67.2)	1524(54.1)	81.592	<0.001
Agreed multiple sex partners	547(11.5)	293(15.0)	254(9.0)	40.507	<0.001
Agreed one night stand	1260(26.4)	575(29.4)	685(24.3)	15.389	<0.001
Agreed extramarital lover	936(19.6)	450(23.0)	486(17.3)	24.301	<0.001
Agreed homosexuality	2885(60.5)	1300(66.5)	1585(56.3)	50.455	<0.001
Agreed condom should be used in premarital sexual behavior	4073(85.4)	1765(90.3)	2308(82.0)	64.335	<0.001
Agreed condoms are the safest measure for preventing STD and unintended pregnancy	2594(54.4)	1265(64.7)	1329(47.2)	142.839	<0.001
**Behaviors**					
Work at place of entertainment (yes)	308(6.5)	179(9.2)	129(4.6)	40.015	<0.001
Has had a boyfriend(yes)	3167(66.4)	1439(73.6)	1728(61.4)	77.694	<0.001
Practice masturbation (yes)	1137(23.8)	429(22.0)	708(25.2)	6.488	0.011
Ever had sexual intercourse (yes)	863(18.1)	468(24.0)	395(14.0)	76.562	<0.001

a Missed cases were excluded.

b Significance tests was chi-squared tests for dichotomous variables.

### Risky sexual behaviors and risks

Table 3 showed the mean age of first sex among only-child was earlier than students with siblings (19.1±1.5 vs. 19.4±2.0). More students with siblings had first sexual experience with men not their “boyfriends” and experienced coercion at first sexual behavior than only-child (6.4% vs. 3.3%; 28.8% vs. 20.7%). However, the proportions of reporting having multiple partners in lifetime or/and in one period among only-child were higher than those among students with siblings (35.9% vs.26.6; 6.8% vs. 3.5%). Also, more only-child (2.4%) had sex with women than students with siblings (0.5%).

**Table 3:** Risky sexual behaviors and risks among sexually active female students who were only-child and students with siblings.

Variables^a^	Total (n = 863)	Only-child			
		Yes (n = 468)	No (n = 395)	*t* or *χ^2b^*	p
**Sexual behavior**					
Mean age of first sex(Mean ± SD)	19.3±1.7	19.1±1.5	19.4±2.0	2.543	0.011
Partner first sex was not boyfriend	40(4.7)	15(3.3)	25(6.4)	4.743	0.029
Coercion first sex	206(24.4)	95(20.7)	111(28.8)	7.387	0.007
Partner last year was not boyfriend	14(2.2)	7(2.0)	7(2.5)	0.219	0.640
Has had multiple lifetime partners	253(31.7)	156(35.9)	97(26.6)	7.901	0.005
Has had multiple partners in one period	41(5.4)	29(6.8)	12(3.5)	4.041	0.044
Had sex with married man	48(5.6)	25(5.3)	23(5.8)	0.094	0.759
Homosexuality	13(1.5)	11(2.4)	2(0.5)	4.910	0.027
**Contraceptive behavior**					
Didn't use a condom first sex	648(79.5)	351(78.7)	297(80.5)	0.396	0.529
Inconsistent condom use during sex in the past 12 years	189(29.7)	103(28.3)	86(31.5)	0.768	0.381
**Risks**					
Self-reported pregnancy	151(17.5)	85(18.2)	66(16.7)	0.314	0.576
Self-reported reproductive infection/STD	176(22.7)	108(24.9)	68(20.0)	2.589	0.108

a Missed cases were excluded.

b Significance tests was chi-squared tests for dichotomous variables.

### The effects of only-child on sex-related acknowledge, attitude and behavior

To examine the relationship between only-child and sex-related acknowledge, attitude and behavior, a multiple logistic regression analyses were performed with only child as independent variables (X), and with 26 sex-related acknowledge, attitude and behavior variables as dependent variables(Y). Social-demographic characteristics such as age, major, grade, nationality, hometown area, and family economic status were adjusted in analyses (Table 4).

**Table 4:** Multivariate analyses assessing the effects of only-child on sex-related acknowledge, attitude and behavior.

Variables	Only-child(Yes)^a^		
	Standardized Regression Coefficient	Standard Error	Test of significance T-test or OR (95%CI)
**Knowledge(n = 4769)**			
Score in Reproduction and Contraception knowledge	0.141	0.209	10.002^ c^
Score in Sexually Transmitted Diseases and AIDS Knowledge	0.122	0.123	8.243^ c^
Score in Sexual and Reproductive Health Knowledge	0.149	0.297	10.448^c^
**Attitudes(n = 4769)**			
Agreed premarital sex	0.293	0.068	1.34(1.17–1.53)^ c^
Agreed multiple sex partners	0.427	0.100	1.53(1.26–1.86)^ c^
Agreed one night stand	0.154	0.073	1.17(1.01–1.35) b
Agreed extramarital lover	0.206	0.081	1.23(1.05–1.44) b
Agreed homosexuality	0.397	0.068	1.49(1.30–1.70)^ c^
Agreed condom should be used in premarital sexual behavior	0.590	0.100	1.81(1.48–2.20)^ c^
Agreed condoms are the safest measure for preventing STD and unintended pregnancy	0.542	0.069	1.72(1.50–1.97)^ c^
**Behaviors(n = 4769)**			
Work at place of entertainment (yes)	0.165	0.139	1.18(0.90–1.55)
Has had a boyfriend(yes)	0.288	0.072	1.33(1.16–1.54)^ c^
Practice masturbation (yes)	−0.046	0.077	0.96(0.82–1.11)
Ever had sexual intercourse (yes)	0.353	0.087	1.42(1.20–1.69)^ c^
**Risky sexual behaviors(n = 863)**			
Mean age of first sex	−0.017	0.111	−0.540
Partner first sex was not boyfriend	−0.810	0.360	0.45(0.22–0.90) b
Coercion first sex	−0.402	0.179	0.67(0.47–0.95) b
Partner last year was not boyfriend	0.225	0.645	1.25(0.35–4.43)
Has had multiple lifetime partners	0.207	0.173	1.23(0.88–1.73)
Has had multiple partners in one period	0.700	0.392	2.01(0.93–4.34)
Had sex with married man	−0.293	0.337	0.75(0.39–1.44)
Homosexuality	1.598	0.828	4.94(0.98–25.03)
Didn't use a condom first sex	−0.202	0.194	0.82(0.56–1.20)
Inconsistent condom use during sex	−0.290	0.194	0.75(0.51–1.10)

a Standardized regression coefficient and odds ratio with 95% confidence interval in parentheses was adjusted for age, major, grade, nationality, hometown area, and family economic status.

b p<0.05 ^c^p<0.01.

Data in table 4 showed that only-child were more likely to have high sex-related acknowledge score, agree with premarital sex, multiple sex partners, one night stand, extramarital lover and homosexuality, have a boyfriend and experience sex than children with siblings. It also appeared that only-child were less likely to experience coercion at first sex and have first sexual intercourse with men not their “boyfriends” than children with siblings.

## Discussion

There is a huge difference in urban and rural areas regarding to China's one-child policy, the existence of significant geographic differences led to the one-child distribution, that most of the only-child have city background, non-only-child mostly have rural background. Some scholars have estimated that the proportion of urban and rural only-child may fluctuate around 6:1 [18]. Our findings also showed similar characteristics, 68.1% of the only-child female students were from capital cities, only 2% of the only-child were from rural areas, and non-only-child from rural areas were up to 28.3%.

Rural and urban areas' differences in education may limit female university students to access sexual knowledge from schools and families [19]. School sex education itself is in the development stage in China; compared to the cities, the backward rural areas are lack of teaching facilities, teachers, lack of information, and also there is the deep-rooted traditional concept – all of which led to the result that adolescent sex education in rural schools is seriously hampered, so that the rural young people have inadequate sex knowledge [20].

On the other hand, in terms of the family sex education, parental education level determines the quality of the implementation of family sex education. Data showed that parents with college background and above conducted sex education to their children having the ratio of 22.31% and 35.17%, respectively, while the parents of junior high school and below have 13.00% and 20.62% [21]. The higher the parents' level of education, and the higher level of knowledge, the more supportive attitude on sex education, whereas rural parents were limited by traditional thinking and level of education, so offered few sex education for their children, this point of view could be supported by the proportion of undergraduates who had not received family sex education (urban 24.6% vs. rural 42.2%) [22]. In addition, the one-child family economic situation is better than non-only child families, so that the only child family's growing environment is different from the non-only-child. Superior economic conditions make the only-child's parents have better conditions to build a family environment, and strive to create a family space that is conducive to the healthy growth of children [23], which can not only further strengthen the parent-child relationship, and also can open only-child's vision, and enhance their social contacts [5], provide more channels to contact information.

Rebecca, etc's African-American youth survey showed that a proper understanding of AIDS knowledge will help them to form healthy sexual awareness and sexual attitudes [24]; Jin Cancan, etc. quantified the relationship between students' sexual knowledge and sexual attitudes, and had pointed out that sexual knowledge score was most closely related to attitude of tolerance, which indicated that the more of the students' knowledge, the more open attitude of one-night stands, extramarital affairs [19]. Our findings showed that female only-child college students with a rich knowledge have more open attitude toward one-night stand, multiple sex partners, premarital sex, and extramarital sex, than non-only-child female students, which is similar to the above studies and other research findings [14], [15].

Early studies found that parents play an important role in the formation of sexual attitudes. Fisher's study showed children's sexual attitudes had a significant relationship with their mother's, and mothers are more likely to talk about sex with her daughter [25]. A survey of young people in China also found that compared to men, women are more likely to communicate with their parents to get adolescence knowledge and sex knowledge [26]. However, Daugherty and Burger reported that the sexual attitudes of boys were easier to be influenced by their parents, the girls' sexual attitudes were more likely to be consistent with that of their peers; they thought that compared to boys, parents were more ashamed to talk about sexual issues with daughter, and girls tend to have intimate friendship with same-sex friends, so to access sexual knowledge from their peers easily [27]. Who played the role in female sexual attitude formation is still controversial, but parents' and peers' impact on individual attitudes was without doubt.

For young adolescents, puberty reverse psychology make teens keep distance from parents but get closer to the peer groups, especially in some sensitive issues, young people often listen to or adopt the opinions or recommendations from companions [28]. Data pointed out that when Chinese college students face sex issues, more than half of the students (55.0%) tend to seek help from classmates and friends, only 10% of the students indicated that they would seek help from the parents [29]. Previous studies have confirmed the absence of brothers and sisters had no adverse affect on social interaction situation of the only-child college students, oppositely, this particular family environment “forcing” only-child college students to interact more often with classmates and peers, this phenomenon can be explained by the “social interaction compensation and diffusion theory” [30]. Today's younger generations' attitudes generally are more open, which created a more tolerable social environment for the opening sex attitudes of only-child. In addition, the core family as an important variable can significantly predict the frequency and extent of the sex communication between children and parents [21]. Growing up in a family of three, the only-child has closer contact with parents and peers than non-only child. Compared with non-only child, their access to sex knowledge and the channels of formation of open attitude are broader, which is the main cause for differences between these two groups in sexual attitudes.

Lefkowitz found that the openness of the sex attitude were closely related to the number of sexual partners [31]; survey carried out in the United States showed that free sexual attitudes prompted young people have younger first sex age [32]; Peng, et al thought that students' sexual knowledge and sexual attitudes had significant positive correlation with sexual behavior [33]. Our survey showed that compared to non-only-child, only-child female college students tend to fall in love more frequently and have premarital sex. This phenomenon was closely related to some of the characteristics of the only-child population. In one-child families, the parents give a lot of attention to the child, and tend to do everything to meet their demands, leading to the result that one-child has a strong dependence [34]. When the only-child enters university, away from the care of their parents, they tend to extend their attachment to their social circle and the strong desire for peer interaction increases, and they try to create a new intimacy and dependency relationship with peers. Heterosexual love not only plays the role of peer communication, but also reaches a compensation effect of intimacy, so the only-child establish the relationship more easily than non-only child [35]. At the same time, the openness of the sexual attitudes of only-child also leads them prone to premarital sex.

Notably, the first sex partner being not boyfriend and coercion first sex was more common in non-only-child female college students. There is a certain amount of internal links between these two high-risk behaviors to some extent, those whose first sex are not with boyfriends are more likely to report coercion first sex. The young people subjected to unwanted sexual behavior will lead to a series of psychological problems such as depression, anxiety, stress, helplessness, despair and even to produce suicidal ideation and behavior, which particularly had deep impact on women [36]. Previous studies have found a harmonious and happy family is important factors to avoid offspring's unwanted sex [37], and maternal attachment plays a key role [38]. Maternal attachment is mothers feel their children occupy an important place in their life. In the non-only-child families, the mother has a number of different interactive objects, and the interactive objects are often different in different time and places, leading to more dispersed mother's attention, so their parent-child interaction frequency are not as high as that of only-child families. Strouse, et al found when mother attachment relationship is weak, girls are more vulnerable to the negative impact of the film, television in terms of sexual contents [39]. Mass media with the obvious gender bias often preached sexual tendencies from the position of the male sexual hedonism, which make inexperienced girls easily misunderstand [40], resulting in an contradictory mind when they interact with the opposite sex, which increases the likelihood of unwanted behavior [41].

From the psychological perspective, both the only-child and non-only child have needs for love, but the study found that non-only-child female students have a lower rate of romantic relationship than those only-child. Possibly because of the non-only-child lack of interpersonal skills, they are more likely to communicate with people through virtual channels. A survey shows the frequency and depth of non-only-child college students' network contacts are higher than the only-child. Non-only-child college students are more likely to communicate with the opposite sex which was 12.8% higher than the only-child college students, and as to communication motivation, the rate of non-only-child college students that selected “looking for romantic love” was also significantly higher than the only-child [42]. Since the Internet is a convenient, hidden channel, it is possible to provide misrepresenting information for users [43], this uncertainty also increase unwanted sexual behavior among the non-only-child in their relations with net friends.

As cultural phenomenon, sexual knowledge and sexual attitudes' impacts on sexual behavior are also constrained by the social and cultural background. The survey showed that the only-child female students scored higher on knowledge of contraception, and also hold a more positive attitude towards condom use compared to non-only-child, however, the two groups had no significant differences in consistent condom use, suggesting that knowledge and attitudes did not alter behavior, which was related to the recognition of gender roles in China's traditional culture. Using a condom during sex often requires consultation and cooperation between the two sides. In China and some Asian countries, there is deep-rooted patriarchal feudal ideology [44], [45], so the women are still in a weak position in the relations between the sexes, easier succumb to the requirements of the male, lack of the right to speak, and ultimately lead to the occurrence of unsafe sex [46]. Therefore, skills-based training should be provided accordingly while providing sex education for women, in addition to the education of the necessary knowledge, attitude, such as how to conduct effective negotiations on condom use with the opposite sex, how to refuse sexual requirements.

Although the only-child female students have a richer sex knowledge and a more open attitude than non-only-child, but except for their love and sexual experience significantly higher than that of non-only-child, the two groups have no significant difference in the risky sexual behaviors, such as having sex at a younger age, multiple sex partners, indicating that the only-child female students' open sexual attitude does not lead to too many risky sexual behaviors. China's survey showed that college students' approval attitude towards premarital sex of themselves was lower than to that of their social network ties [19]. The reason for this phenomenon is that there are significant differences in the requirements for sex attitudes and behaviors of men and women, which is called “sexual double standard” [47]. Deng Xin-mei, et al carried out a investigation of students in both Guangzhou and Hong Kong on premarital sexual behavior, extramarital affairs, one-night stands, etc., and found that there were significant double standard, most respondents think that the above acts are more appropriate for men [48], suggesting that China ancient concept of female chastity and ideological tolerance of male sexual behavior is still having an impact on college students. In a qualitative research on the sexual attitudes of college students in China, a lot of girls believed that virgin is still an important condition for male mate selection criteria [49]. This also shows that compared to foreign countries, sexual culture in China is still relatively conservative, especially for women, traditional social customs and ethics still restricted their sexual behaviors [50]. This is also consistent with previous domestic studies [51].

Sexual knowledge, sexual attitudes, and sexual behavior are not one-way influencing factors; rich sex knowledge and open sexual attitudes would also be influenced by sexual behavior. The survey in United States found that college students had more sex knowledge if they had sex experience [52]; a study in China's Yunnan got similar results: sexually experienced female university students' knowledge was significantly higher than non-sexual experienced ones, and their sexual attitudes also significantly more open than the non-sexual experienced students [53]. The reason for above phenomenon may be that the students with sexual experience concerned for contraception, so they actively seek for sex knowledge. Due to the limitations of this study's method, we can not explore how sexual knowledge, sexual attitudes and behavior influenced each other, which will be a focus for follow-up study.

### Conclusion

Because of different growth environment and education background, the only-child female students have richer sexual knowledge, more open attitudes and more likely to have a boyfriend and engage in sexual intercourse than non-only-child. However, it was more likely for non-only-child to experience first sex with non-boyfriend and/or coercion first sex. Possibly due to relatively conservative social and cultural background in China, only-child who agreed with some risky sexual behaviors (multiple sex partners, one night stand, etc) did not tend to practice these behaviors; only-child holding more approval attitude to condom use was not more likely to use condoms consistently compared with non-only-child. These findings suggested that sex education for female students should be designed according to different requirement of only-child and non-only-child. For non-only-child, necessary sexual knowledge, attitude and skills of refusing sexual requirements need to be stressed. While other skills in sexual practice such as how to conduct effective negotiations on condom use with the opposite sex should be provided not only for non-only-child but for only-child.
